# Krill oil extract suppresses the proliferation of colorectal cancer cells through activation of caspase 3/9

**DOI:** 10.1186/s12986-019-0382-3

**Published:** 2019-08-17

**Authors:** Abilasha Gayani Jayathilake, Elif Kadife, Rodney Brain Luwor, Kulmira Nurgali, Xiao Qun Su

**Affiliations:** 1 Institute for Health and Sport, Victoria University, P.O. Box 14428, Melbourne, 8001 Australia; 2 Department of Surgery, The Royal Melbourne Hospital, The University of Melbourne, Parkvill, Australia; 3 Department of Medicine, Western Health, The University of Melbourne, St Albans, Australia; 4 Regenerative Medicine and Stem Cells Program, Australian Institute for Musculoskeletal Sciences, Melbourne, Australia

**Keywords:** Krill oil extract, Eicosapentaenoic acid, Docosahexaenoic acid, Human colorectal cancer cells, Caspase 3/9

## Abstract

**Background:**

Currently available treatments for colorectal cancer (CRC) associate with numerous side-effects that reduce patients’ quality of life. The effective nutraceuticals with high anti-proliferative efficacy and low side-effects are desirable. Our previous study has reported that free fatty acids extract (FFAE) of krill oil induced apoptosis of CRC cells, possibly associated with changes in mitochondrial membrane potential (MMP). The aims of this study were to compare the anti-proliferative efficacy of FFAE from krill oil on CRC cells with commonly used chemotherapeutic drug, Oxaliplatin, and to investigate the molecular mechanisms underlying the anti-proliferative effects of krill oil with a focus on intrinsic mitochondrial death pathway.

**Methods:**

Three human CRC cell lines, including DLD-1, HT-29 and LIM-2405, and one mouse CRC cell line, CT-26, were treated with FFAE of KO and the bioactive components of krill oil, eicosapentaenoic acid (EPA) and docosahexaenoic acid (DHA) for 24 h and 48 h. Similarly, these cell lines were treated with Oxaliplatin, a commonly used drug for CRC treatment, for 24 h. The effects of FFAE of KO, EPA, DHA and Oxaliplatin on cell proliferation, mitochondrial membrane potential and reactive oxygen species (ROS) were determined via WST-1, JC-10, and ROS assays respectively. The expression of caspase-3, caspase-9 and DNA damage following treatments of FFAE of KO was investigated via western blotting and immunohistochemistry.

**Results:**

The FFAE of KO, EPA and DHA significantly inhibited cell proliferation and increased formation of ROS in all four cell lines (*P <* 0.01). A small dose of FFAE from KO ranged from 0.06 μL/100 μL to 0.12 μL/100 μL containing low concentrations of EPA (0.13–0.52 μM) and DHA (0.06–0.26 μM) achieved similar anti-proliferative effect as Oxaliplatin (*P >* 0.05). Treatments with the FFAE of KO, EPA and DHA (2:1 ratio) resulted in a significant increase in the mitochondrial membrane potential (*P* < 0.001). Furthermore, the expression of active forms of caspase-3 and caspase-9 was significantly increased following the treatment of FFAE of KO.

**Conclusions:**

The present study has demonstrated that the anti-proliferative effects of krill oil on CRC cells are comparable with that of Oxaliplatin, and its anti-proliferative property is associated with the activation of caspase 3/9 in the CRC cells.

## Introduction

Colorectal cancer (CRC) is the third most common cancer diagnosed and the fourth leading cause of cancer-related death affecting both men and women worldwide [1, 2]. The initiation of CRC is a complex and multifactorial process that involves progressive accumulation of genetic and epigenetic alterations, and these cause the normal colonic/rectal mucosa transformation into invasive metastatic carcinoma [3, 4] The risk factors associated with the development of CRC include consumption of processed and red meat, sedentary life style, obesity, smoking and alcohol intake [5, 6]. Currently available treatments for CRC include surgery, chemotherapy and radiotherapy [7], with surgery being more effective when the disease is diagnosed at the early stage. However, in most cases CRC is diagnosed at the advanced stages (III or IV) when tumour has already spread to other parts of the body [8]. The available treatments for the later stages of CRC are chemotherapy and radiotherapy, which have numerous side-effects that impact patient’s quality of life [9, 10]. In recent years more attention has been devoted to nutraceuticals as alternative and/or conjunctive therapeutic agents for cancer prevention and treatment [10, 11].

Long chain omega-3 poly unsaturated fatty acids (LC n-3 PUFA), eicosapentaenoic acid (EPA, 20:5n-3) and docosahexaenoic acid (DHA, 22:6n-3), derived from fish and other seafood, have been reported to inhibit the proliferation and development of several cancers including the CRC [12, 13]. Epidemiological studies have shown that populations consuming large amounts of LC n-3 PUFA-rich fish oil have a lower risk of CRC [14]. In vitro studies have found that EPA and DHA exert their effects on cancer cells via several mechanisms including changing the membrane composition, altering intracellular Ca^++^ concentrations as well as intracellular pH, modifying mitochondrial membrane potential/permeability, changing cellular resistance to ROS damage, and by direct actions on DNA and gene expression [15–23]. Animal studies have also shown that fish oil supplementation reduced the number and size of tumours, angiogenesis and metastasis [24–28].

Human consumption of fish-derived food products has increased steadily, and the global capture of fish will become un-sustainable in the future. Krill, a shrimp-like marine zooplankton, has been identified as an alternative source due to its wide and abundant distribution [29]. The main commercially available krill oil is extracted from Antarctic krill (*Euphasia superba*), living in the Southern Ocean, and it has become an important source of LC n-3 PUFA in the last decades [29]. One of the advantages of krill oil compared to fish oil is that it has a high concentration of phospholipids and krill oil derived EPA and DHA are mainly bound to those lipids, predominantly phosphatidylcholine [29, 30] while in the fish oil they are bound to the triglycerides [31, 32]. Previous studies have suggested that the LC n-3 PUFA from phospholipids may penetrate through the cell membrane more efficiently, therefore lead to a higher bioavailability [32] and more health benefits.

To date, only few in vitro studies have investigated the anti-proliferative effect of krill oil [11, 33, 34], Su et al. [11] have reported that krill oil inhibited cell proliferation in 43B and SJSA-1osteosarcoma cells. Zhu et al. [33] have shown the inhibitory effects of krill oil on SW-480 CRC cell line. In the previous study, we have observed that FFAE of krill oil significantly inhibited the proliferation and induced apoptosis of human CRC cell lines HCT-15, SW-480 and Caco-2 [34]. We also found that the pro-apoptotic property of krill oil may be related to the increase in mitochondrial membrane potential (MMP) [34]. Based on these findings, we hypothesized that change in MMP of CRC cells following the treatment with krill oil would cause the release of cytochrome *c*. That would then activate caspase-9 and caspase-3 and lead to nuclear DNA damage thus apoptosis of CRC cells. The aims of this study were to compare the anti-proliferative efficacy of FFAE of krill oil on CRC cells with a chemotherapeutic drug, Oxaliplatin, commonly used for CRC treatment. Furthermore, we investigated the molecular mechanisms associated with the anti-proliferative effects of krill oil, with a focus on intrinsic mitochondrial death pathway.

## Materials and methods

### Cell lines and culture conditions

The human colon adenocarcinoma cell lines DLD-1 and HT-29; and mouse colon cancer cell line CT-26 were obtained from the American Tissue Culture Collection (ATCC), Manassas, VA,USA (Catalogue No.CCL-221, HTB-38 and CRL-2638), and human colon cancer cell line LIM-2405, was obtained from the Ludwig Institute for Cancer Research, Melbourne, Australia (Catalogue No.CBA-0165). All cell lines were maintained in RPMI1640 medium (Sigma Aldrich, Castle Hill, NSW) supplemented with foetal calf serum (FCS, 10%) (Hyclone Quantum Scientific, Clayton South, VIC), glutamine (10 mM), 4–2-hydroxyethyl-1-piperazineethanesulfonic acid, sodium pyruvate (10 mM) and penicillin (100 U/mL)/ streptomycin (100 μg/mL) (Sigma Aldrich, Castle Hill, NSW). Cells were grown at 37 °C in 5% CO_2_ humidified atmosphere. Exponentially growing cells that were > 90% viable were used for assays.

### Extraction of free fatty acids from krill oils and fatty acid preparation

Free fatty acids were extracted from the krill oil (Swisse Wellness Pty Ltd., Victoria, Australia) following the hydrolysis (saponification) method of Salimon et al. [35]. The extracts were dissolved in 100% ethanol and stored at -20 °C. The final treatment solutions contained < 0.1% ethanol as a solvent. Individual EPA and DHA were purchased from Nu-Chek-Prep, Elysian, USA (Catalogue No. T-325 and A-662). Fatty acid solutions were made up by dissolving the individual fatty acids in ethanol and the final treatment solutions contained < 0.1% ethanol as a solvent.

### Cell proliferation assay

A water-soluble tetrazolium-1 (WST-1) assay kit (Roche Diagnostics GmbH, Germany) was used to determine the proliferative potential of cancer cells. Cells were seeded and cultured at 1 × 10^4^ cells per well in 96-well plates for 24 h and then treated with EPA or DHA solutions for 24 and 48 h or FFAE of krill oil for 24 h. All treatments were performed in triplicates and the concentrations of EPA, DHA and Oxaliplatin were selected based on their respective dose-response curves. Four concentrations (50 μM, 100 μM, 200 μM and 250 μM) of DHA and three concentrations (50 μM, 100 μM, 200 μM) of EPA were used. FFAE of KO were diluted in ethanol at three concentrations: 0.03 μL, 0.06 μL and 0.12 μL/100 μL prior to the treatment and that equate to the concentrations of EPA and DHA per 100 μL well at 0.13/0.06, 0.26/0.13 and 0.52/0.26 μM, respectively. In all experiments, 0.1% ethanol was used as a vehicle control, non-treated cells as a negative control, and Oxaliplatin as a positive control.. The WST-1 reagent (10 μL) was added to each well after respective treatment time point and incubated at 37 °C for 1 h. Cell proliferation was measured using a micro-plate reader (Varioskan Flash, Thermo Scientific) at the absorbance of 450 nm. Each experiment was repeated three times for each cell line.

### Reactive oxygen species (ROS) assay

The generation of ROS in the mitochondria after each treatment was assessed using MitoSOX™ Red mitochondrial superoxide (Invitrogen, Australia). The cells were seeded in 96-well plates at a density of 5 × 10^4^ cells/well. Cells were treated by the FFAE of KO at 0.12 μL/100 μL dilution, EPA at 200 μM and DHA at 250 μM respectively for 24 h. A working solution of MitoSOX™ was prepared fresh and diluted in the phosphate buffered saline (PBS) in the dark. MitoSOX™ (100 μL) was added to each well and cells were incubated at 37 °C for 40 mins in a shaker with gently shaking (Unimax 1010). The fluorescence intensity was measured using a microplate reader (Varioskan Flash, Thermo Scientific) at excitation/emission (Ex/Em) wavelengths 495/525 nm and Ex/Em 490/595 nm. The amount of ROS generated in the mitochondria was measured as a ratio of aggregate (Em 525 nm) to monomeric forms (Em 595 nm) of MitoSOX™. Three replicates for each treatment and two individual experiments were performed.

### Mitochondrial membrane potential (MMP) JC-10 assay

Cells were seeded at 5 × 10^4^ cells/well in clear bottom 96-well plates (Corning TM Costar TM 3603, USA) and incubated for at 37 °C for 24 h prior to undergoing the following treatments for 24 h: EPA at 200 μM, DHA at 250 μM, FFAE of KO at 0.12 μL/100 μL and six combinations of EPA and DHA in a ratio of 1:1 and 2:1 at the concentrations of 50 μM, 100 μM, 200 μM of EPA and DHA (for example, three mixtures of a ratio of 1:1 containing 50 μL EPA and 50 μL DHA at concentrations of 50 μM or 100 μM, or 200 μM. Similarly, a 2:1 ratio containing 66.67 μL of EPA and 33.33 μL of DHA at concentrations of 50 μM or 100 μM, or 200 μM). The final volume of the combined mixture was 100 μL. The MMP was measured using the JC-10 assay kit (ab 112,134, Abcam, Australia) as per the manufacturer’s instructions. Briefly, 50 μL of JC-10 reagent was added to each well after the treatment and incubated at 37 °C for 1 h in the dark. Thereafter, 50 μL of assay buffer B was added. Fluorescent intensity was measured using a microplate reader (Varioskan Flash, Thermo Scientific) at Ex/Em = 485/520 nm and Ex/Em = 540/570 nm. The change of mitochondrial membrane potential was measured as the ratio of aggregate (Em 520 nm) to monomeric forms (Em 570 nm) of JC-10. The increase in the ratio indicates the mitochondrial membrane depolarisation. Three replicates were performed for each treatment. The results were verified through at least three individual experiments.

### Immunocytochemistry

Cells were grown on chamber slides (Ibidi, Australia) at a density of 1 × 10^4^ cells/well in 8-well plates and incubated at 37 °C in a 5% CO_2_ environment for 24 h. They were then treated with the FFAE of KO at 0.12 μL/100 μL for 8 h. Cells were fixed with 4% paraformaldehyde for 10 mins before permeabilizing for 15 mins with 0.1% Triton X-100 PBS. The donkey serum (10%) in PBST was added before incubation at room temperature for 1 h to block the endogenous activity. Then CRC cells were incubated at 4 °C overnight followed by staining with the primary antibodies for cleaved caspase-3 (1:500, rabbit mAb 9664 (ASP 175 (5A 1E), Cell Signalling Technologies, MA, USA) and for the DNA/RNA damage (1:500, mouse monoclonal anti-DNA/RNA damage antibody (15A3), Abcam, MA, USA). The expression of cleaved caspase-9 was investigated by staining with rabbit anti- cleaved caspase-9 mAb [1:500 (ASP 330 - E5Z7N), Cell Signalling Technologies, MA, USA]. The cells were washed with PBS (3 × 10 mins) before incubating with secondary antibodies (diluted to 1:250) labelled with different fluorophores: Alexa Fluor 594-conjugated donkey anti-rabbit (Jackson Immuno Research Laboratories, PA USA) and Alexa Fluor 488-conjugated donkey anti-mouse (Jackson Immuno Research Laboratories, PA USA) at room temperature for 2 h. All these antibodies have been diluted in PBS with 2% donkey serum and 0.01% Triton X-100. Then the cells were washed with PBS 3 × 10 mins and incubated for 2 mins with the fluorescent nucleic acid stain, 4′-6-diamidino-2-phenylindole (DAPI). Finally, all cells were washed with PBS for 10 mins before mounting on the fluorescent mounting medium (DAKO, USA). Cell images were taken with the Eclipse Ti confocal laser scanning system (Nikon, Tokyo, Japan). The excitation wavelengths for FITC and Alexa Fluor 594 were adjusted to 488 nm and 559 nm respectively. Each fluorophore was measured using 8 images taken at 20x magnification with a total area of 2 mm^2^. All images were then calibrated to standardize for a minimum basal fluorescence and converted to binary. Fluorescence intensity was measured using Image J software (National Institute of Health, USA). The results were verified through at least three individual experiments.

### Western blot

The expression of pro and active caspase-3 and caspase-9 proteins were investigated in two cell lines, DLD-1 and HT-29. Cells were treated by FFAE of KO at 0.03 μL and 0.12 μL for 1 h, 4 h, 8 h and 12 h and the results were compared with the ethanol control. After the treatment, cells were collected and lysed in the radioimmunoprecipitation assay buffer (RIPA buffer) (pH 7.4, 150 mM NaCl, 0.1% SDS, 0.5% sodium deoxycholate, 1% NP-40 in PBS, Sigma) containing a protease and phosphatase inhibitors cocktail (Roche Applied Science, USA). Cellular proteins (12 μg) from each sample were separated using 4 to 20% sodium dodecyl sulphate (SDS)/polyacrylamide gel electrophoresis. The separated fragments were transferred to 0.22 μm polyvinylidene fluoride membranes, which were blocked with 5% skim milk in PBST (0.1% Tween-20) by incubating at room temperature for 90 mins in a 40 RPM speed shaker. The membrane was allowed to react with primary antibodies against caspase-3 (1:1000, rabbit, E87 (ab32351), Abcam, MA, USA) and caspase - 9 (1:1000, rabbit E23 (ab32539), Abcam, MA, USA) overnight at 4 °C. Membrane was washed three times in PBST (0.1% Tween-20) and incubated with secondary antibody goat anti-rabbit IgG H&L horseradish peroxidase (HRP) (Abcam, ab6721, MA, USA) at room temperature for 1 h. Again, the membrane was washed three times in PBST (0.1% Tween-20). Glyceraldehyde-3-phosphate de-hydrogenase (GADPH) (Abcam, ab9485, MA, USA) was used as the loading control. The protein detection was performed using enhancing chemiluminescence reagents (Clarity™ Western ECL Substrate, Bio-Rad, USA). Chemiluminescence signals were captured using the FUSION FX System (USA). The expression level of each protein was quantified using Fusion Capt advance FX7 software. The results were verified through at least three individual experiments.

### Statistical analysis

All data were analysed using SPSS 22 software (IBM, USA). Mixed model ANOVA was used to determine the significance between treatments. The significance of repeated measure at different time point was analysed using one-way ANOVA. Post-hoc analysis was conducted using Tukey HSD test for multiple comparisons. *P* < 0.05 was considered as significant. The results were expressed as mean ± SD in tables or mean ± SEM in figures.

## Results

### Effect of FFAE of krill oil on the proliferation of CRC cell lines compared with Oxaliplatin

**Fig. 1:**
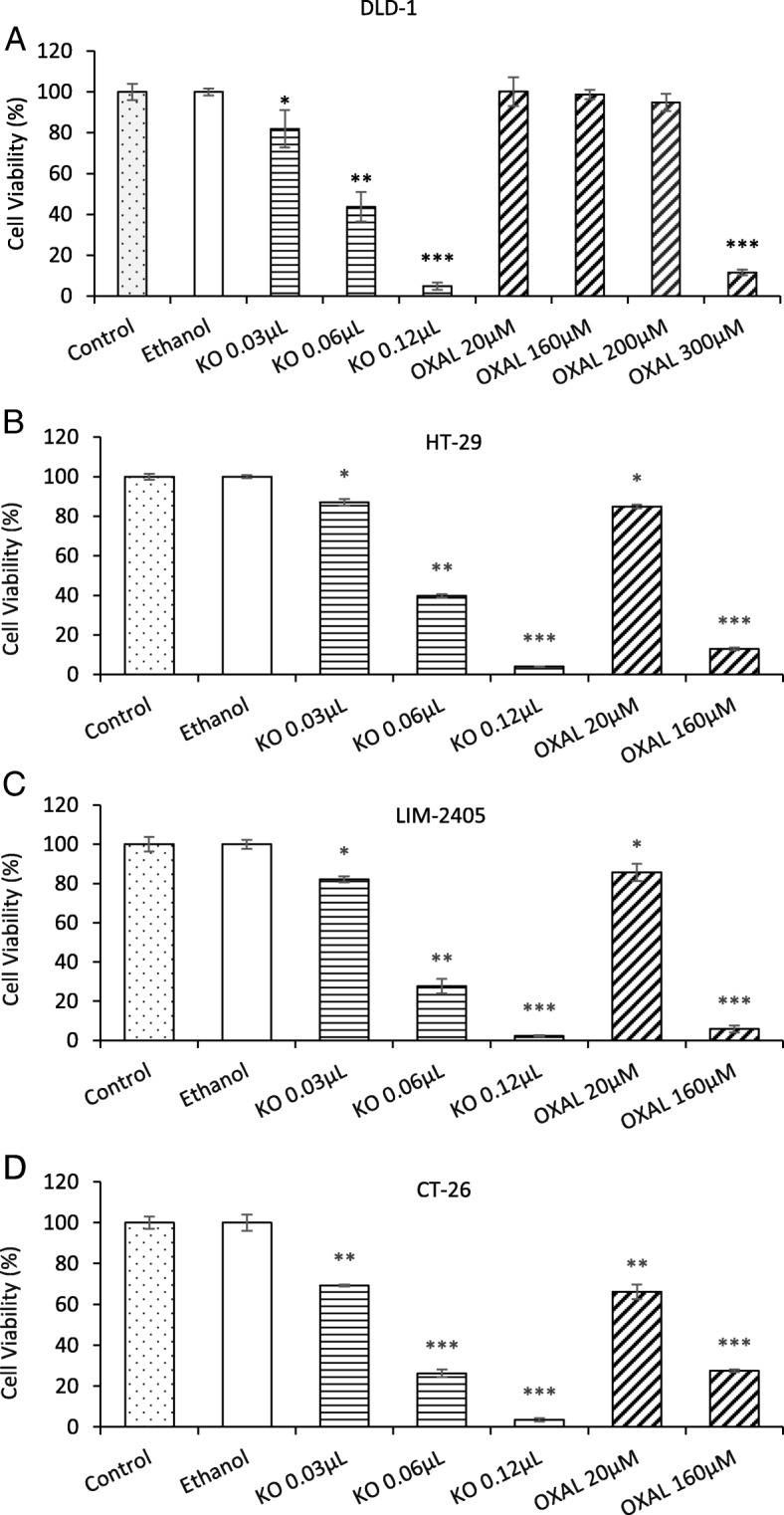
Effects of FFAE of krill oil on the proliferation of CRC cells compared to the anti-cancer drug Oxaliplatin. Cell viability of DLD-1 (**a**), HT-29 (**b**), LIM-2405 (**c**) and CT-26 (**d**) cells were determined using WST-1 assay following 24 h of treatment with FFAE of krill oil (KO) at the concentrations of 0.03 μL/100 μL (containing 0.13 μM EPA/0.065 μM DHA), 0.06 μL/100 μL (containing 0.26 μM EPA/0.13 μM DHA), and 0.12 μL/100 μL (containing 0.52 μM EPA/0.26 μM DHA) or chemotherapeutic drug, Oxaliplatin (OXAL). The experiment was repeated three times for each cell line. Data are expressed as mean ± SEM (*n* = 3), *p < 0.05, **p < 0.01 and ***p < 0.001 indicate a significant difference between the treatment and Ethanol (vehicle) control

DLD-1, HT-29, LIM-2405 and CT-26 cancer cells were treated with the FFAE of krill oil at the concentrations of 0.03 μL, 0.06 μL, and 0.12 μL/100 μL for 24 h. The cell proliferation of treatment groups was compared to that of ethanol (vehicle control) treated cells (Fig. 1). Treatments with FFAE of krill oil at concentrations of 0.03–0.12 μL/100 μL for 24 h have significantly reduced proliferation of DLD-1 cells by 18.2 ± 7.5% (*P* < 0.05) and up to 95.2 ± 1.8% (*P* < 0.001). Treatment with lower doses of Oxaliplatin did not show significant effect on the highly resistant DLD-1 cells while high dose of Oxaliplatin (300 μM) reduced cell proliferation by 88.5 ± 1.4% (*P* < 0.001) (Fig. 1a).

Similarly, FFAE of krill oil inhibited cell proliferation of HT-29 and LIM-2405 cells following 24 h of treatment. At the low dose (0.03 μL/100 μL) of krill oil FFAE, cell proliferation of HT-29 and LIM-2405 reduced by 12.9 ± 1.7% (*P <* 0.05) and 17.9 ± 1.5% (*P* < 0.05) respectively, compared to vehicle control cells (Fig. 1b and c). The greatest impact on proliferation was observed at the 0.12 μL/100 μL dose of FFAE, with significant 95.9 ± 0.1% reduction for HT-29 cells (*P* < 0.001) and 97.7 ± 2.3% for LIM-2405 cells (*P* < 0.001) (Fig. 1b and c). Treatment with Oxaliplatin at the concentrations of 20 μM and 160 μM for 24 h has resulted in significant inhibition of proliferation with 15.1 ± 0.9% and 87.1 ± 0.7% reductions for HT-29 cells (Fig. 1b) and14.3 ± 4.4 (*P* < 0.05) and 94.1 ± 1.8 (*P* < 0.001) reductions for LIM-2405 cells (Fig. 1c).

The CT-26 mouse CRC cells were more sensitive to krill oil FFAE treatment, compared to human cell lines (Fig. 1d). The cell proliferation was reduced by 30.7 ± 0.4% (*P* < 0.01) at the 0.03 μL/100 μL concentration of krill oil FFAE, and by 96.5 ± 0.8% (*P* < 0.001) at the 0.12 μL/100 μL concentration. These results were also comparable to the effects of Oxaliplatin treatments (Fig. 1d).

### Effects of n-3 PUFA on cancer cell proliferation

**Fig. 2:**
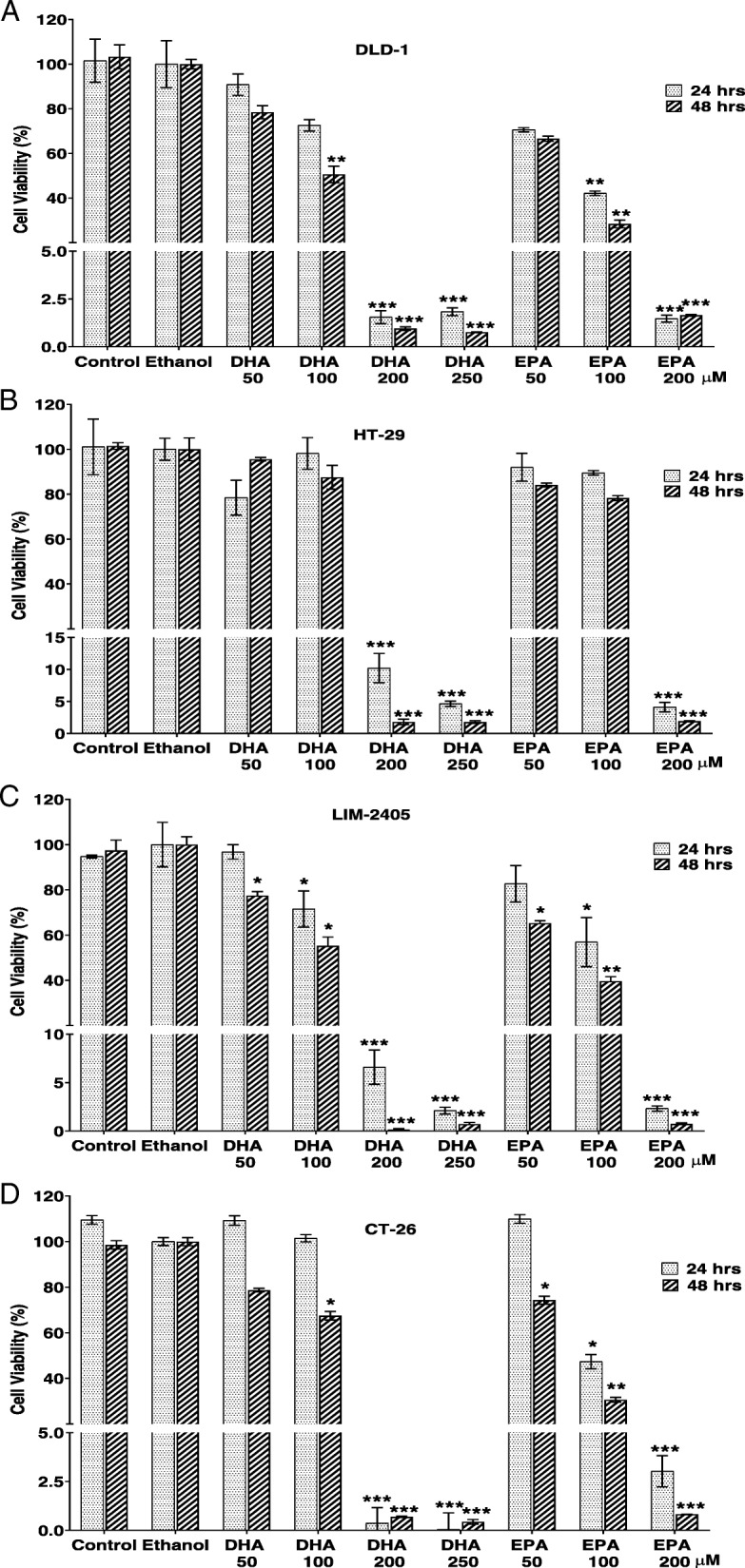
Proliferation of CRC cells following treatment with EPA and DHA. Cell viability of DLD-1 (**a**), HT-29 (**b**), LIM-2405 (**c**) and CT-26 (**d**) cells were determined using WST-1 assay following treatment with DHA and EPA for 24 and 48 h. The experiment was repeated three times for each cell line. Data are expressed as mean ± SEM (*n* = 3), *p < 0.05, **p < 0.01 and ***p < 0.001 indicate a significant difference between the treatment and Ethanol (vehicle) control

The effects of n-3 PUFA, DHA and EPA, on proliferation of human colon cancer cells (DLD-1, HT-29 and LIM-2405) and mouse colon cancer cell (CT-26) are shown in Fig. 2. Both DHA and EPA have inhibited proliferation of all four cell lines in a dose-dependent manner.

DLD-1 cells treated with DHA and EPA at concentrations less than 100 μM did not cause significant changes in cell proliferation, compared to vehicle treated cells at 24 and 48 h time points (Fig. 2a). The 100 μM dose of DHA reduced cell proliferation by 49.4 ± 3.2% (*P* < 0.01) following 48 h of treatment. DHA treatments at 200 μM and 250 μM concentrations significantly inhibited cell proliferation by more than 95% at both time points (*P* < 0.001 for both). The low concentration of EPA (100 μM) has resulted in significant reduction of cell proliferation by 57.9 ± 0.9% at 24 h and 71.7 ± 1.5% at 48 h (*P* < 0.01 for both). Treatment with higher concentration of EPA (200 μM) has inhibited cell proliferation by 98.5 ± 0.2% at both time points (*P* < 0.001 for both).

Treatments with lower concentrations (50 μM and 100 μM) of both DHA and EPA have not shown significant effects on HT-29 cells (Fig. 2b). Treatments with 200 μM and 250 μM of DHA reduced cell proliferation by 89.8 ± 2.0% and 95.4 ± 0.4% respectively at 24 h and 98.2 ± 0.4% and 98.2 ± 0.2% at 48 h (*P* < 0.001 for all). Treatment with 200 μM EPA inhibited cell proliferation by 95.9 ± 0.6% at 24 h and 98.1 ± 0.1% at 48 h (*P* < 0.001 for both).

As shown in Fig. 2c, LIM-2405 cells treated with lower concentrations (50–100 μM) of DHA have shown significantly inhibited cell proliferation at both 24 and 48 h (*P* < 0.05). Treatments with DHA at 200 μM and 250 μM resulted in marked reduction of cell proliferation (93.4 ± 1.5% and 99.3 ± 0.1% respectively) (*P* < 0.001 for both). Treatments with EPA at concentrations of 50–200 μM have inhibited cell proliferation significantly (*P* < 0.05 for all). Remarkable results were observed at the high concentration (200 μM) with a reduction of 97.7 ± 0.2% and 99.3 ± 0.1% cell proliferation being recorded at 24 and 48 h respectively (*P* < 0.001 for both).

The lower concentration of DHA (50 μM) did not show significant effect on proliferation of CT-26 cells. The proliferation of CT-26 cells was reduced significantly by DHA at the concentrations of 200 μM and 250 μM (98.9 ± 0.1% - - 99.9 ± 0.7%) (*P* < 0.001 for all), (Fig. 2d).

Treatments with 50 μM of EPA reduced the proliferation of CT-26 cells slightly at 48 h (*P* < 0.05) while 100 μM of EPA showed stronger inhibitory effect (*P* < 0.05). Treatment with 200 μM EPA inhibited most of cell proliferation with a reduction of 96.9 ± 0.7% at 24 h and 99.2 ± 0.0% at 48 h (*P* < 0.001 for both).

### Effect of FFAE of krill oil, EPA and DHA on ROS formation

**Fig. 3:**
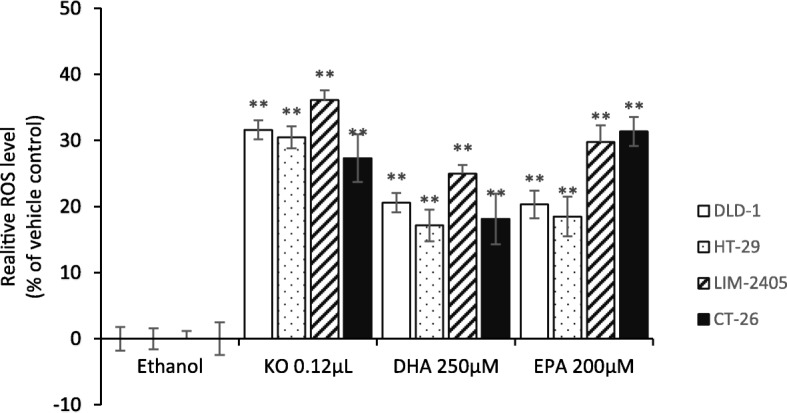
ROS formation in mitochondria of CRC cells after 24 h of treatment with FFAE of krill oil, EPA and DHA. The mitochondrial superoxide level was measured using the MitoSox™ and was presented as a percentage comparison to the ROS level in Ethanol (vehicle) treated cells. Three replicates for each treatment and two individual experiments were performed. Data are expressed as mean ± SEM (*n* = 3). **p < 0.01 indicates a significant difference between the treatment and Ethanol (vehicle) control

The effects of FFAE of krill oil, EPA and DHA on ROS formation in all four cell lines are shown in Fig. 3. After 24 h of KO treatment ROS production increased by approximately 30% across all CRC cell lines, compared to the vehicle treated cells. Although to a less degree than KO, both DHA and EPA resulted in similar trend of increased ROS production with approximately 20–30% of increase in all four cell lines following EPA treatment and 20–25% increase following DHA treatment.

### Effect of FFAE of krill oil, EPA and DHA on mitochondrial membrane potential of cancer cells

**Fig. 4:**
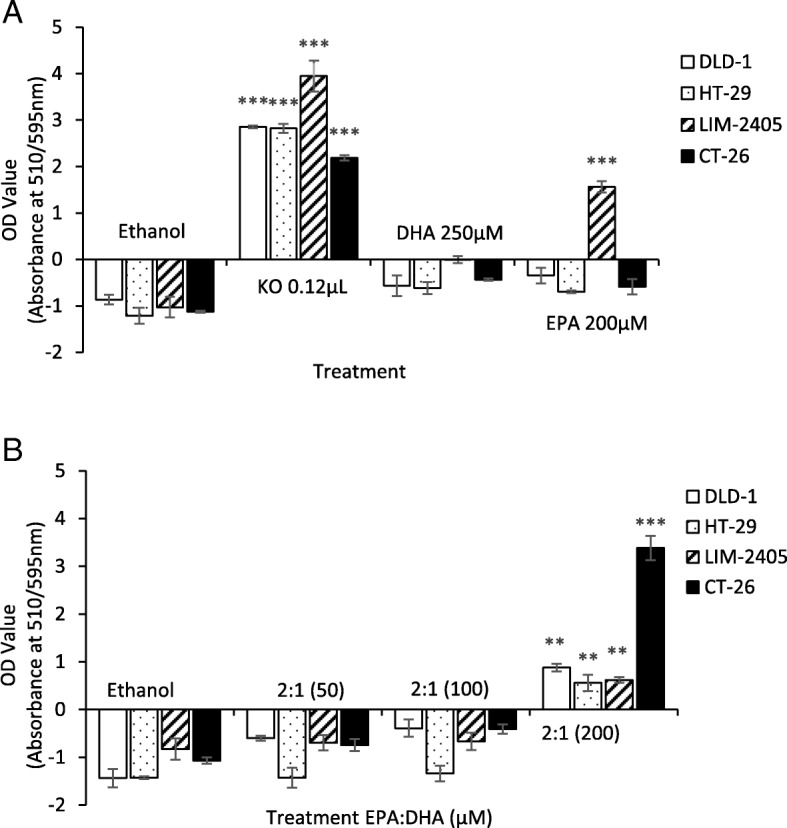
Mitochondrial membrane potential (MMP) in CRC cells following treatment with FFAE of krill oil, EPA and DHA. (**a**) MMP of DLD-1, HT-29, LIM-2405 and CT-26 cells was measured using the JC-10 fluorescent MMP microplate assay following 24 h of treatment with FFAE of krill oil (0.12 μL/100 μL, containing 0.52 μM EPA/0.26 μM DHA), DHA (250 μM) or EPA (200 μM). (**b**) Effects of treatment with combined EPA and DHA at a 2:1 volume ratio. Three replicates for each treatment and two individual experiments were performed. Data are expressed as mean ± SEM (*n* = 3), **p < 0.01 and ***p < 0.001 indicate a significant difference compared to Ethanol (vehicle) control

Changes of mitochondrial membrane potential in all four CRC cell lines following treatments by krill oil FFAE at 0.12 μL/100 μL, DHA at 250 μM and EPA at 200 μM for 24 h are shown in Fig. 4a. Significant MMP depolarisation was observed across cell lines following the treatment with krill oil FFAE compared to the ethanol control (*P* < 0.001) (Fig. 4a). However, no significant changes were observed following treatments by EPA or DHA alone except in LIM-2405 cells (*P* < 0.001). There were also no significant changes in MMP in any of the four cell lines following 24 h of treatment with a mixture of EPA and DHA in a volume ratio of 1:1 at the concentrations of 50 μM, 100 μM and 200 μM (data not shown). However, a significant increase of MMP was observed in all four CRC cell lines following treatments with combined EPA and DHA in a volume ratio of 2:1 at the concentration of 200 μM (*P* < 0.01 for all). Treatments at lower concentrations (50 μM and 100 μM) with 2:1 volume ratio of EPA and DHA did not have any significant effect on MMP (Fig. 4b).

### Expression of caspase-3 and caspase-9 levels following treatments of FFAE from krill oil

**Fig. 5:**
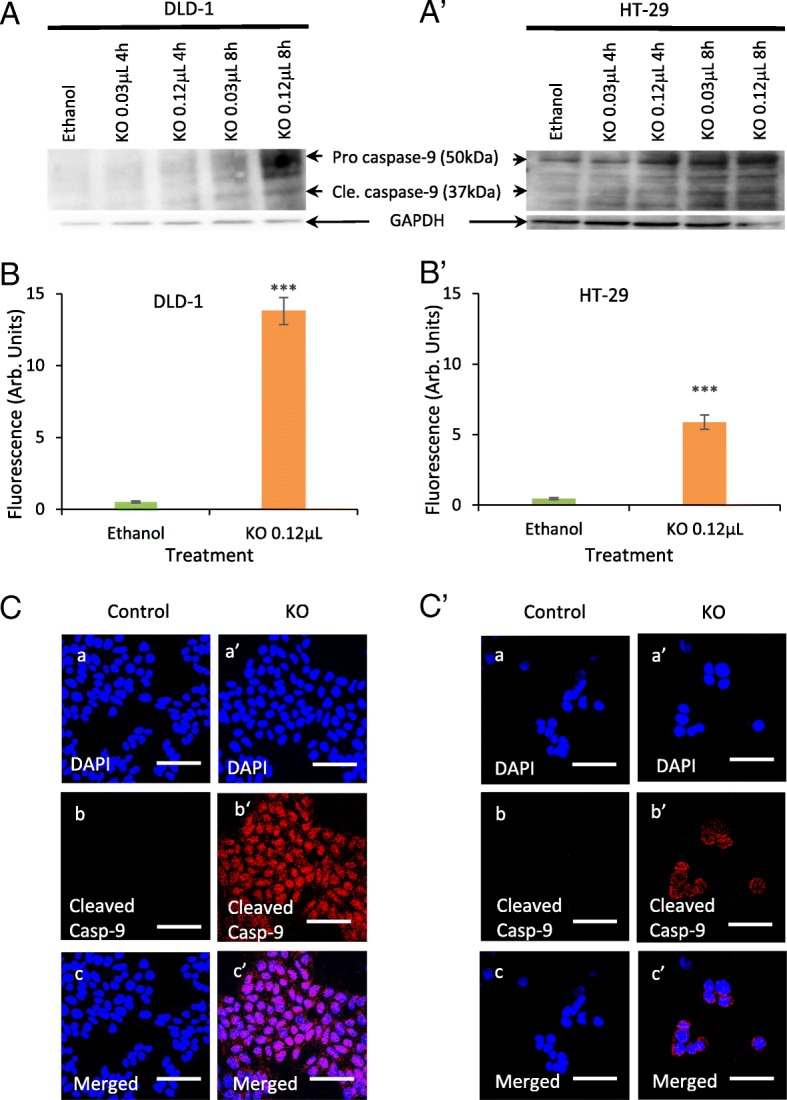
Activation of caspase-9 in CRC cells after treatment with FFAE of krill oil. The expression of caspase-9 and cleaved caspase-9 was measured by western blotting in DLD-1 (A) and HT-29 (A’) cells following treatment with FFAE of krill oil at 0.03 μL/100 μL (containing 0.13 μM EPA/0.065 μM DHA) and 0.12 μL/100 μL (containing 0.52 μM EPA/0.26 μM DHA) for 4 h and 8 h. Fluorescent intensity of subcellular distribution of cleaved caspase-9 in DLD-1 (B-C) and HT-29 (B′-C′) cells was determined using a monoclonal antibody for cleaved caspase-9 following 8 h of treatment with FFAE of krill oil at 0.12 μL/100 μL(containing 0.52 μM EPA/0.26 μM DHA). Scale bar = 50 μM. Magnification = 60X. The results were verified through at least three individual experiments. Data are expressed as mean ± SEM. ***p < 0.001 compared to Ethanol control

**Fig. 6:**
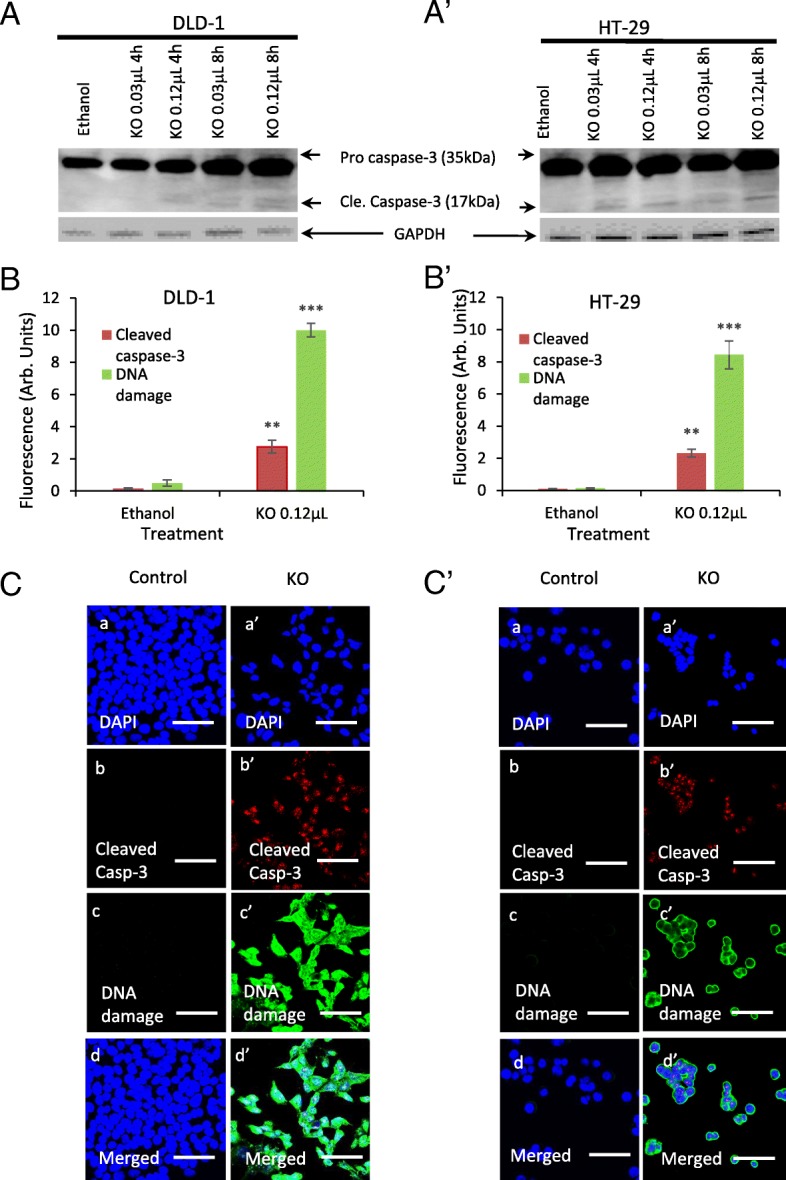
Activation of caspase-3 in DLD-1 and HT-29 cells following treatment with FFAE of krill oil. The expression of caspase-3 and cleaved caspase-3 was measured by western blotting in DLD-1 (A) and HT-29 (A’) following treatment with FFAE of krill oil at 0.03 μL/100 μL (containing 0.13 μM EPA/0.065 μM DHA) and0.12 μL/100 μL (containing 0.52 μM EPA/0.26 μM DHA) for 4 h and 8 h. Fluorescent intensity of subcellular distribution of cleaved caspase-3 and DNA damage in DLD-1 (B-C) and HT-29 (B′-C′) cells was determined using monoclonal antibodies for cleaved caspase-3 and DNA/RNA damage (anti-8-OHdG) following 8 h of treatment with FFAE of krill oil at 0.12 μL/100 μL (containing 0.52 μM EPA/0.26 μM DHA) . Scale bar = 50 μM. Magnification = 60X. The results were verified through at least three individual experiments. Data are expressed as mean ± SEM. **p < 0.01 and ***p < 0.001 compared to Ethanol control

The expression of caspase-3 and caspase-9 proteins was investigated via western blotting and immunohistochemistry, and data from DLD-1 and HT-29 cells were shown in Figs. 5 and 6. Low and high doses of krill oil FFAE (0.03 μL/100 μL and 0.12 μL/100 μL) were selected for treatments. The extraction of proteins was carried out at 2, 4, 8 and 12 h following treatments. The level of caspase-3 and caspase-9 started to increase after 4 h of treatment and declined after 12 h, therefore, only the results obtained from 4 and 8 h are presented.

**Fig. 7:**
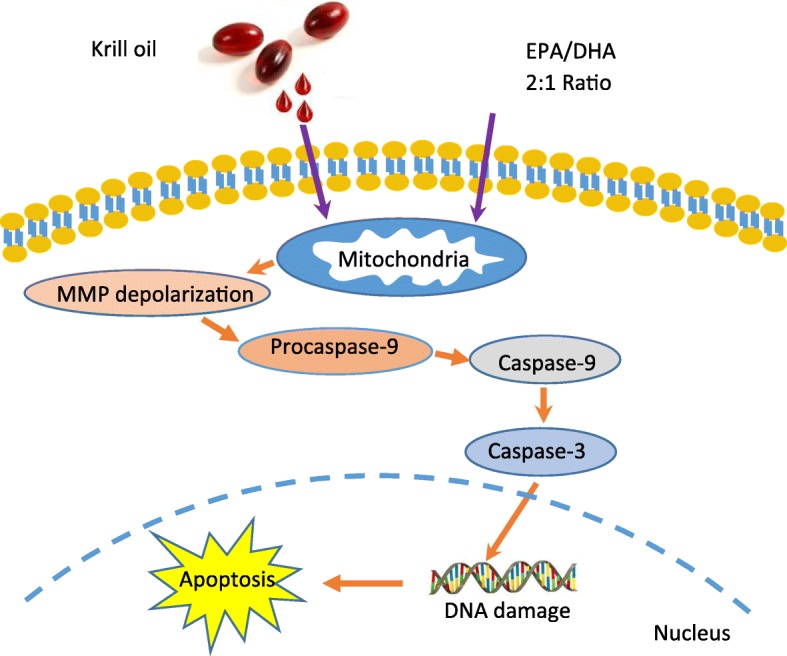
Schematic summary of the death signaling pathways initiated by the FFAE of krill oil in DLD-1 and HT-29 cells. The FFAE of krill oil and a combination of EPA/DHA exert their effects on cancer cells by changing the mitochondrial membrane potential (MMP). That results in the activation of caspase-9 and caspase-3 and lead to nuclear DNA damage hence possible apoptosis of cancer cells

The FFAE of krill oil has activated caspase-9 and resulted in increase of cleaved caspase-9 in DLD-1 and HT-29 cells at 4 and 8 h of treatments. DLD-1 cell line treated by krill oil FFAE at the concentration of 0.03 μL/100 μL and 0.12 μL/100 μL has shown an increase in the protein expression by 6.8 and 22.2% at 4 h and 43.5 and 95.7% at 8 h respectively compared to ethanol control (Fig. 5A). Similar increase in caspase-9 protein level was observed in HT-29 cells after treatment with krill oil FFAE by 1.5 and 49.4% at 4 h and by 73.7 and 84.2% at 8 h respectively compared to ethanol control (Fig. 5A’). The immunohistochemistry results were consistent in both cell lines after 8 h of treatment with 0.12 μL/100 μL of krill oil FFAE. A significant increase in the fluorescence intensity of cleaved caspase-9 was observed in both DLD-1 and HT-29 cells following treatments with krill oil FFAE compared to the ethanol control (*P* < 0.001) (Fig. 5B-B’). The number of cells showing cleaved caspase-9 immunofluorescence was higher in DLD-1 and HT-29 cells treated with FFAE of krill oil compared with vehicle control group (Fig. 5C-C’).

The FFAE of krill oil has activated caspase-3 and resulted in increase of cleaved caspase-3 in both cell lines in a similar way as to caspase-9. DLD-1 cells treated by krill oil FFAE at the concentrations of 0.03 μL/100 μL and 0.12 μL/100 μL have shown an increase in the expression of caspase-3 by 4.6 and 29.1% at 4 h and by 65.7 and 92.9% at 8 h respectively (Fig. 6A). Increased in caspase-3 protein level was observed in HT-29 cells after treatment with krill oil FFAE at the concentration of 0.03 μL/100 μL by 37.9% at 4 h and concentration of 0.12 μL/100 μL by 89.3% at 8 h (Fig. 6A’). These results were further verified by the immunohistochemistry assay (Figs. 6B-B’, C-C′).

The DNA damage following treatment with FFAE of krill oil at a concentration of 0.12 μL/100 μL was also assessed using immunohistochemistry (Fig. 6B-B’, C-C′). It was found that the level of DNA damage was enhanced significantly (*P* < 0.001) in both DLD-1 and HT-29 cell lines following treatments with FFAE of krill oil for 8 h compared to ethanol control.

## Discussion

The present study investigated the effects of krill oil FFAE on human CRC cell lines DLD-1, HT-29, LIM-2405 and a mouse CRC cell line CT-26. The results demonstrated that krill oil FFAE significantly inhibited the growth of all four cell lines and confirmed the anti-proliferative property of krill oil on other CRC cell lines and osteosarcoma previously reported by us [11, 34] and others [33]. The anti-proliferative effects of krill oil FFAE were similar to the effects of its bioactive constituents, EPA and DHA although the effective dose of krill oil extract is much lower. This indicates that the anti-proliferative properties of krill oil are attributed to EPA and DHA. The reason that a lower dose of krill oil extract (0.13–0.52 μM EPA/0.065–0.26 μM DHA) achieved similar effect as isolated EPA (50–200 μM) and DHA (50–250 μM) may be related to the fact that krill oil contains not only EPA and DHA but also a range of other fatty acids including saturated fatty acids (SFA) and monounsaturated fatty acids (MUFA), and the interactions between SFA, MUFA and EPA and DHA may have enhanced the efficacy of these n-3 fatty acids, as reported by Dias et al. [36] and MacDonald-Wicks and Garg [37]. In addition, we found that a relatively low dose of krill oil FFAE could achieve a remarkable anti-proliferative effect comparable with Oxaliplatin, a commonly used clinical drug for CRC treatment [38]. Furthermore, this study provides evidence for the possible mechanistic pathway involved in the anti-proliferative effects of krill oil. Mitochondria appear to be the main target of krill oil FFAE, and the change in MMP have resulted in the activation of caspase-9 and caspase-3. This then induced DNA damage leading to apoptosis (Fig. 7).

Previous studies suggested that EPA and DHA, alone or in combination, have the ability to suppress CRC through changing the membrane fluidity, anti-inflammation and altering signalling pathways involved in carcinogenesis, angiogenesis and cell metastasis [39–42]. Several molecular mechanisms underlying the anti-cancer effect of these fatty acids have been proposed. These include the inhibition of cell proliferation and promotion of apoptosis through the tumour suppressor (Hippo) pathway [13]; suppression of pro-inflammatory molecules, prostaglandin PGE_2_, an eicosanoid derived from arachidonic acid (AA) through the COX-2 pathway [43, 44]; promoting cell death via altering the mitochondrial membrane potential [19] and EGFR complex, as well as associated intracellular signalling pathways involving ERK 1/ 2, and mechanistic target rapamycin (mTOR) [45]. Furthermore, EPA and DHA have been reported to downregulate anti-apoptotic proteins and lead to the activation of caspase pathways [46–49].

Caspases play a significant role in the apoptosis of cancer cells. These enzymes are activated through three pathways including the extrinsic death receptor, intrinsic mitochondrial, and intrinsic endoplasmic reticulum (ER) death pathways [50–52]. The present study suggests that the anti-proliferative property of krill oil is closely associated with the intrinsic mitochondrial death pathway initiated by changes in the MMP. This process involves the change of mitochondrial outer membrane permeability/depolarisation due to DNA damage or ROS accumulation. Membrane depolarisation causes the release of cytochrome *c* into the cytosol. The cytochrome *c* is involved in the formation of pro-caspase-9 and apoptotic protease activating factor-1 (APAF-1) complex that activate executioner caspase-3 or 7 through initiator caspase-9 [52–55]. Previous studies have reported that the release of cytochrome *c* is associated with proteins of Bcl-2 family involved in the signal transduction and various cytotoxic stimuli [56]. The interaction of Bcl-2 proteins regulates the integrity of outer mitochondrial membrane (OMM). The pro-apoptotic Bcl-2 proteins change the permeability of mitochondrial membrane that allows the release of cytochrome *c* from the mitochondrial intermembrane space into the cytosol. Cytochrome *c* is directly involved in the activation of caspase-3 pathway via the apoptosome complex that contains cytochrome *c*/APAF-1/caspase-9 [55]. The caspase-9 in the apoptosome complex recruits caspase-3 into the apoptosome complex [57] to produce many cellular and biochemical events involved in apoptosis [58]. Therefore, the activation of caspases is essential for cancer suppression [59]. The present study has demonstrated the changes in the MMP and activation of caspase-9 and caspase-3 in CRC cells following the treatment of krill oil FFAE. We also observed the significantly high level of DNA damage in all four cell lines compared to ethanol (control) treatment. This finding agrees with the study by Giros et al. [19] demonstrating that EPA and DHA induce apoptosis through the intrinsic death pathway in colon cancer cells Caco-2, HT-29, SW-480 and HCT-116.. The activation of intrinsic pathway of apoptosis with EPA and DHA treatments have also been reported in human neuroblastoma cells [53] and in multiple myeloma cells [60].

The reactive oxygen species (ROS) have a dual role in cancer development. On the one hand, ROS can promote pro-tumorigenic signalling, facilitating cancer cell proliferation, survival, and adaptation to hypoxia. On the other hand, ROS can promote anti-tumorigenic signalling and trigger oxidative stress–induced cancer cell death [61]. In the present study we found a significant increase of ROS level in CRC cells following treatments by the FFAE of krill oil, EPA and DHA correlated with anti-proliferative effects. Furthermore, we have shown that the FFAE of krill oil is more potent in increasing ROS in the cancer cells than EPA or DHA alone (Fig. 3). In agreement with our study, previous studies on human non-small cell lung cancer (NSCLC) and prostate cancer cell lines, PC3 and DU145, found that DHA induced cellular apoptosis through the over-production of ROS in the mitochondria, which caused inactivation of the PI3K/Akt pathway inhibiting growth and proliferation of cancer cells [62, 63]. In addition, Kang et al. (2010) observed that EPA and DHA increased production of ROS that causes apoptosis of MCF-7 breast cancer cells [64].

ROS are produced in different subcellular regions by the action of different enzymes [65]. Mitochondria produce a large amount of ROS as a by-product of fatty acid metabolism and oxidative phosphorylation during the synthesis of ATP [63, 66]. Our results have shown a significant depolarization of mitochondrial membrane of the CRC cells following the treatment of krill oil FFAE. Furthermore, a combination of EPA and DHA at 200 μM in a ratio of 2:1 also resulted in a significant depolarization of mitochondrial membrane while a combination of EPA and DHA at 200 μM in 1:1 ratio has not shown significant effect on the MMP. In our previous study [34] we also observed a significant increase of MMP in CRC cell lines HCT-15, SW-480 and Caco-2 after treatment by krill oil FFAE but not by EPA or DHA alone. However, So et al. (2015) and Giros et al. (2009) reported that EPA and DHA treatments alone at the concentrations between 50 and 60 μM altered the MMP and resulted in apoptosis of human neuroblastoma and CRC cell lines Caco-2, HT-29 and SW-480 [19, 67]. The discrepancy between our study and that by So [67] and Giros et al. [19] could be due to the sensitivity of different CRC cells. It is known that ROS production during catabolism of long chain (LC) fatty acids may reflect a complex process. LC fatty acid-induced ROS production at the physiological range of MMP and relative insensitivity to the changes of MMP has been previously reported [68]. A mitochondrial membrane potential-independent ROS production has been observed in brain mitochondria [69, 70]. Several factors might contribute to the increased ROS production without changes in MMP. LC fatty acid breakdown can generate intermediates and by-products that can inhibit the mitochondrial electron transport chain to potentially augment ROS production. The mitochondrial ROS load also depends upon the activity of antioxidant processes. It has been suggested that some components of the mitochondrial glutathione antioxidant system are inhibited during LC fatty acid catabolism [68]. Further studies are warranted to uncover the mechanisms of ROS production following treatment of individual EPA and DHA.

Oxaliplatin is a commonly used chemotherapeutic drug for patients with metastatic CRC (stage III and IV) [71]. However, it has been reported to cause severe side-effects including cytopenia, peripheral neurotoxicity, nausea, vomiting, diarrhoea and constipation [72, 73]. The present study has demonstrated that the anti-proliferative effects of krill oil on CRC cells are comparable with that of Oxaliplatin. Based on the findings from this study, the equivalent human dose of treatment would be 302.45 mg of EPA, and 328.48 mg of DHA. This is equivalent to 4–5 krill oil capsules (1 g) daily. This dose is feasible in practice. More importantly krill oil is safe as no associated side-effects have been reported [29, 32].

## Conclusions

This study demonstrated that the FFAE of krill oil has a remarkable anti-proliferative property, comparable with that of Oxaliplatin. This is likely attributed to its bioactive components, EPA and DHA. The pro-apoptotic effects of krill oil, EPA and DHA on CRC cells appear to be associated with intrinsic mitochondrial death pathway. The treatment with FFAE of krill oil resulted in a significant increase in ROS and MMP. This then activated caspase-9 and caspase-3 leading to DNA damage and cellular apoptosis. The outcome of this study implicates a possible clinical application of krill oil. Further animal studies and human clinical trials are required to validate the efficacy of krill oil on the prevention and treatment of CRC.

## Data Availability

The datasets from the present study are available from the corresponding author upon request.

## Abbreviations

AA: Arachnoid Acid
Akt: protein kinase
APAF-1: apoptotic protease activating factor
ATCC: American Tissue Culture Collection
Bak: pro-apoptotic Bcl-2 proteins
Bcl-2: B cell lymphoma protein
COX-2: cyclo-oxygenase-2
CRC: Colorectal cancer
DAPI: 4,6-diamidino-2-phenylindole
DHA: docosahexaenoic acid
DMEM: Dulbecco’s Modified Eagle’s Medium
DMSO: Dimethyl Sulfoxide
EPA: eicosapentaenoic acid
ER: endoplasmic reticulum
FCS: Foetal Calf serum
FFA: Free Fatty Acids
FFAE of KO: Free Fatty Acids Extractions of Krill Oil
GAPDH: Glyceraldehydes-3-phosphate de-hydrogenase
HEPES: 4–2- hydroxyethyl − 1-piperazineethanesulfonic
LC n-3 PUFA: Long chain n-3 PUFA
MMP: Mitochondrial Membrane Potential
OXAL: Oxaliplatin
PBS: Phosphate Buffered Saline
PBS-T: Phosphate Buffered Saline + Tween 20
PGE2: Prostaglandin E2
PGE3: Prostaglandin E3
PUFA: Polyunsaturated Fatty Acids
RIPA: Radioimmunoprecipitation assay buffer
ROS: Reactive oxygen species
SD: Standard Deviation
SDS: Sodium dodecyl Sulphate
SEM: Standard error of mean
WST-1: Water soluble tetrazolium-1
